# Randomized, Double-Blinded, Multicenter, Placebo-Controlled Trial of Shenfu Injection for Treatment of Patients with Chronic Heart Failure during the Acute Phase of Symptom Aggravation (Yang and Qi Deficiency Syndrome)

**DOI:** 10.1155/2019/9297163

**Published:** 2019-02-25

**Authors:** Xianliang Wang, Zhiqiang Zhao, Jingyuan Mao, Tinghai Du, Yuanping Chen, Hao Xu, Nan Liu, Xiaolong Wang, Jianguang Wu, Rong Li, Yong Xu, Yingqiang Zhao, Lei Wang, Jingsong He, Junhua Zhang, Jingbo Zhai, Guoyuan Zhao, Yazhu Hou, Shuai Wang, Chunxiang Liu

**Affiliations:** ^1^First Teaching Hospital of Tianjin University of Traditional Chinese Medicine (TCM), Tianjin, 300193, China; ^2^The First Affiliated Hospital of Henan University of T.C.M., Henan, 450000, China; ^3^The First Affiliated Hospital of Guangxi University of Chinese Medicine, Guangxi, 530000, China; ^4^Xiyuan Hospital, China Academy of Chinese Medical Sciences (CACMS), Beijing, 100091, China; ^5^The First Affiliated Hospital of Guangzhou University of T.C.M, Guangzhou, 510450, China; ^6^Department of Cardiovascular, Shuguang Hospital Affiliated to Shanghai University of Traditional Chinese Medicine, Shanghai, 201203, China; ^7^Affiliated Hospital of Jiangxi University of T.C.M., Jiangxi, 330000, China; ^8^Teaching Hospital of Chengdu University of T.C.M., Chengdu, 610000, China; ^9^Second Affiliated Hospital of Tianjin University of T.C.M., Tianjin, 300143, China; ^10^West China Hospital of Sichuan University, Sichuan, 610000, China; ^11^Ruikang Hospital Affiliated to Guangxi University of Chinese Medicine, Guangxi, 530000, China; ^12^Evidence-Based Medicine Center, Tianjin University of Traditional Chinese Medicine, Tianjin 300193, China

## Abstract

**Background:**

Shenfu injection (SFI) has shown a remarkable therapeutic effect in patients with chronic heart failure (CHF) during the acute phase of symptom aggravation since it became commercially available in 1987. However, the therapeutic effect of SFI has not been validated in a standard clinical study. As a pilot clinical trial, this study aimed to evaluate the safety and efficacy of SFI for treatment of CHF patients during the acute phase.

**Methods:**

A total of 160 patients experiencing acute phase CHF were enrolled in this study and randomly assigned to receive the placebo (placebo group, 150 ml glucose (GS)) or SFI (SFI group, 50 ml SFI + 100 ml GS) in addition to their standard medications for CHF treatment. The treatment lasted for 7 ± 1 days, and the follow-up continued for 28 ± 3 days after treatment. The primary endpoints were New York Heart Association (NYHA) classification and Traditional Chinese Medicine (TCM) syndrome scores.

**Results:**

After 7±1 days of treatment, the efficacy of SFI according to improvements in NYHA and TCM syndrome scores in the SFI group (78.38% and 89.19%, respectively) was significantly higher than that in the placebo group (61.43% and 60.00%, respectively; P<0.05). The SFI group had a longer increase in amplitude than the placebo group (113.00 m versus 82.99 m, P<0.05). The incidence of adverse events and other safety indices showed no significant differences between the two groups.

**Conclusion:**

SFI combined with conventional therapy for treatment of CHF during acute symptom aggravation ameliorated the cardiac dysfunction and clinical symptoms and improved the patients' quality of life without any significant AEs compared with the conventional therapy alone.

## 1. Introduction

Chronic heart failure (CHF) is the leading cause of hospitalization in the aged population worldwide and negatively affects the quality of life of patients [1]. CHF patients commonly experience a vicious disease cycle: “hospitalization—improvement—discharge—rehospitalization.” Acute CHF is the most common type of acute heart failure, and, among patients with acute heart failure, the hospital mortality rate ranged from 4% to 11% [2–4], the vascular rehospitalization rate within 30 days was 7%–16% [5], and the mortality rate was 11.3% [6].

Based on the theory of Traditional Chinese Medicine (TCM), the major cause of heart failure is heart Yang deficiency that results from Qi inadequacy and blood stasis. Some traditional Chinese herbs have demonstrated safety and efficacy for the management of heart failure in either animal models or humans. For example, velvet antler of deer ameliorated cardiac dysfunction associated with heart failure in a rat myocardial model with heart failure following myocardial infarction [7], and Qili qiangxin capsules, another TCM, has been shown to be a beneficial auxiliary treatment for heart failure [8].

Shenfu injection (SFI) is a specific TCM extracted from two types of herbs, ginseng radix et rhizome, and radix aconiti carmichaeli. SFI has been shown to exhibit a variety of pharmacological activities, including elevating blood pressure and potentiating myocardial contractility, and has been used for many years to treat patients with cardiovascular diseases in China, such as dilated cardiomyopathy [9], heart failure [10], and acute myocardial infarction [11]. However, there have been few studies performed as a standard clinical trial to validate clinical observations of the safety and efficacy of SFI for treatment of heart diseases. In this pilot randomized multicenter, double-blinded placebo-controlled clinical trial, we aimed to assess the safety and efficacy of SFI for treatment of CHF during acute aggravation.

## 2. Patients and Methods

### 2.1. Study Protocol

This study was a randomized, double-blinded, multicenter, placebo-controlled trial. A total of 160 patients at 11 clinical research centers in China were registered for this study (Supplemental Table 1). The study protocol was approved by the ethics committee of the First Teaching Hospital of Tianjin University of Traditional Chinese Medicine, and other ethics committees at the research centers that participated in this study. This pilot trial was published in* Trials* in May of 2015 [12]. All participants signed an informed consent form. This study was performed in compliance with the Declaration of Helsinki and has been registered in the Chinese Clinical Trial Register (http://www.chictr.org.cn; Register number: ChiCTR-TRC-12002857.

### 2.2. Inclusion and Exclusion Criteria

The inclusion criteria were as follows: (1) according to the diagnostic criteria defined for coronary heart disease, patients had occluded myocardial infarction with a stricture rate of the coronary artery main branch (at least 1 branch) of more than 50%, regardless of percutaneous coronary intervention (PCI) or coronary artery bypass grafting (CABG) therapy; (2) according to the diagnostic criteria of CHF during acute aggravation, patients had a left ventricular ejection fraction (LVEF) ≤50% as determined by the improved Simpson method [13, 14] with one or more of the following: (i) stable cardiac function but sudden fatigue with no reason, (ii) difficulty breathing while doing physical work, or occasionally at night, and had to use a pillow to elevate the position of the head when sleeping, (iii) sudden serious expiratory dyspnea, orthopnoea, agitation, and/or fear; (iv) left ventricle being larger than before; basic heart rate being faster than before (+15–20 times/min); Traube's bruit, (dry) moist crackles, and wheezing rale in lungs especially in the bottom of lungs; (3) meeting the TCM criteria for yang and qi deficiency, which has the main symptoms of palpitations, shortness of breath (dyspnea), and fatigue; (4) age between 40 and 79 years; (5) New York Heart Association (NYHA) classification of III~IV; and (6) signature on informed consent form.

The exclusion criteria were as follows: (1) acute coronary syndrome, acute myocardial infarction within 6 months, revascularization or intention to undergo revascularization within 6 months, cardiogenic shock, serious arrhythmias, cardiomyopathy, rheumatic valvular heart disease, myocarditis, constrictive pericarditis, and pulmonary embolism within a week; (2) severe liver and renal insufficiency (glutamic oxalacetic transaminase [ALT] ≥3 times normal upper limit, creatinine [Cr] ≥3 mg/dL); (3) severe primary disease in the endocrine and hematopoietic system; (4) intention to become pregnant or breastfeed; (5) psychopath; (6) allergy to SFI; (7) participation in other trial within the previous 3 months; (8) life expectancy <3 months as judged by the investigator; and (9) failure to fulfill or obey the study guidelines as judged by the investigator.

### 2.3. Interventions

After application of the inclusion and exclusion criteria, a total of 160 patients were finally enrolled in our study and randomly assigned to two groups: the placebo group, in which 80 patients were given the placebo (GS 150 ml), and the SFI group, in which 80 patients were treated with SFI (SFI 50 ml + GS 100 ml). Patients in both groups simultaneously received their usual care and medications such as diuretics, angiotensin converting enzyme inhibitors (ACEIs), or angiotensin II receptor blockers (ARBs), *β*-receptor blocker, aldosterone-receptor blocker, digoxin, and vasodilator substance prescribed for CHF by the attending physicians. The study medication was uniformly labeled by the sponsor, blinded by the statistics unit, and then sent to each center. Special drug administrators were established in each subcenter, and the researchers informed the nurse after random access to the drug number. The nurse was responsible for preparing the medicine, confecting solution, and serving the transfusion to patients with a disposable optical infusion device. Therefore, the study was not blinded to the nurse but to the researchers, patients, and statisticians. The participants were given SFI or placebo once a day during the 7 (±1)-day course of treatment. Each patient's health status was evaluated before and after treatment including curative indices and safety indices, and all patients were followed up for 28 ± 3 days after administration of the medication by telephone or outpatient visit. The follow-up information included cardiovascular events and rehospitalization (Supplemental Figure 1).

### 2.4. Endpoints

The primary endpoints were NYHA classification and TCM syndrome scores. The secondary endpoints included Lee's CHF scores, 6-minute walk distance (6MWD), LVEF, and the incidence rate of cardiovascular events and heart failure emergency/rehospitalization. The safety endpoints included tests of blood, urine, stool, the levels of serum K, Na, Cl, ALT, aspartate aminotransferase (AST), blood urea nitrogen (BUN), and Cr, and the occurrence of adverse events (AEs)/adverse drug reactions (ADRs).

### 2.5. Efficiency Standard

The efficiency standard was formulated in reference to “the principle of clinical research on treating heart failure with new Chinese Medicine [15]” (2002).

*Heart Function Efficiency Standard*

  Excellent: heart failure was essentially ameliorated or the NYHA classification increased by at least 2 levels.
  Valid: NYHA classification increased by 1 level.
  Invalid: NYHA classification remained the same before and after the treatment.
  Worsened: NYHA classification decreased by at least 1 level.

*Efficiency Standard for Yang and Qi Deficiency Syndrome*

  Excellent: Clinical symptoms were markedly improved, and the TCM syndrome score decreased by 70% compared with that before treatment.
  Valid: Clinical symptoms were improved, 30% ≤ TCM syndrome score < 70%.
  Invalid: Clinical symptoms were not improved.
  Worsened: Clinical symptoms were worsened. The TCM syndrome score increased.

*Lee's Heart Failure Score*

  Excellent: Scores decreased by 75% compared with that before treatment.
  Valid: 50% ≤ decreased scores < 75%.
  Invalid: 0 ≤ decreased scores < 50%.
  Worsened: Decreased scores < 0.

*Sample Size Calculation*. This study was a pilot clinical trial. The sample size was calculated based on a 20% expulsion rate in a clinical study and an expert's opinion. Hence, we recruited 160 patients to this study, who were subsequently allocated at a 1:1 ratio to the SFI and placebo groups.

### 2.6. Statistical Analysis

All statistical analyses were performed at an independent institute—Tianjin Institute of Traditional Chinese Medicine Engineering—with SAS software, version 9.1.3. Data were analyzed according to the full analysis set principle. Continuous variables are presented as the mean ± standard deviation (SD). The comparability of the characteristics between the two study groups was assessed using a two-sample Student t-test for continuous variables and the chi-square test or Wilcoxon test, when appropriate, for categorical variables. The Wilcoxon paired signed-rank test was used for within-group comparisons. The statistical difference in the AE incidence rate between the two study groups was assessed using *χ*^2^ test (or Fisher exact test if not appropriate). A P value <0.05 was considered statistically significant, and all tests were two-tailed.

## 3. Results

### 3.1. Study Population

**Figure 1:**
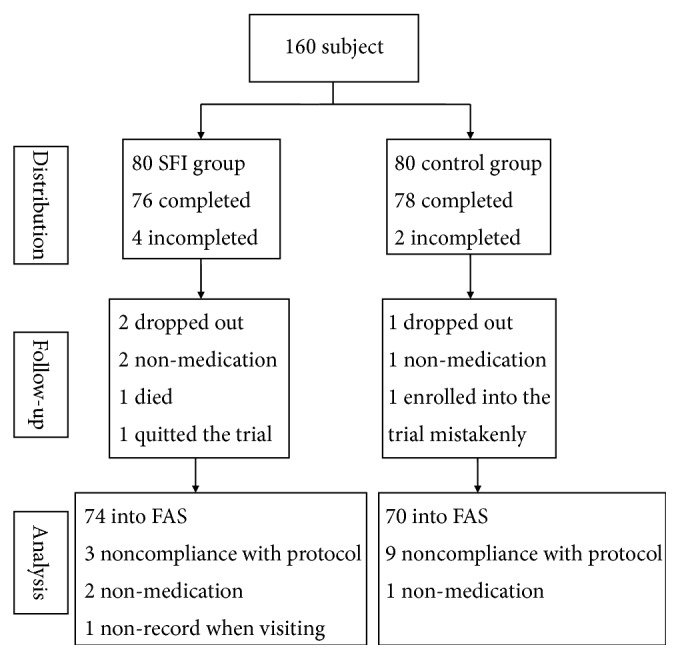
Flow chart of patient selection and study design.

From April 18, 2013, to August 2, 2015, a total of 160 patients were enrolled in this study and randomly assigned to the placebo and SFI groups at a 1:1 ratio. Among these participants, 154 were followed up for 28 ± 3 days after the treatment (76 in the SFI group and 78 in the placebo group); 144 were enrolled in the full analysis set (FAS) (74 in the SFI group and 70 in the placebo group); and 137 were enrolled in the per-protocol set (PPS) (71 in the SFI group and 66 in the placebo group). Medication compliance of all patients was recorded (102.32% in the SFI group and 101.22% in placebo group; Figure 1).

### 3.2. Comparison of Demographic and Baseline Clinical Characteristics of Participants between the SFI and Placebo Groups

The recorded demographic and clinical characteristics included age, gender, medical history, course of heart failure in the acute aggravation phase, information of background medication, NYHA classification, TCM syndrome score, Lee's heart failure score, 6MWD, and LVEF. The distributions of the demographic and clinical characteristics between the SFI and placebo groups were well balanced and homogeneous (P*>*0.05.  Table 1), except that the average heart rate in the SFI group was significantly higher than that in placebo group (P<0.05, Table 1).

### 3.3. Comparison of Primary Endpoints between SFI and Placebo Group

SFI treatment significantly improved the NYHA classification by 78.38% compared to the 61.43% increase observed in the placebo group (P=0.0026, relative risk [RR] = 1.2759, 95% confidence interval [CI]: 1.0231–1.5913; Table 2). Similarly, SFI treatment exhibited a high efficacy for improving the TCM syndrome score (by 89.19%) compared to the 60.00% increase observed in the placebo group (P<0.001, RR=1.4865, 95%CI: 1.2085–1.8285; Table 3).

### 3.4. Comparison of the Secondary Endpoints between the SFI and Placebo Groups

SFI treatment had a significantly higher effective rate (70.27%) for improving Lee's heart failure score compared with placebo treatment (52.17%; P=0.0262, RR=1.3468, 95%CI: 1.0280–1.7646; Table 4). Also, SFI treatment greatly increased the 6MWD (113.00 m) compared with that in the placebo group (82.99 m, P=0.0281; Table 5). Although patients in the SFI group tended to have better LVEF than those in the placebo group, no statistical difference was observed (P>0.05; Table 6). No composite cardiac events (CCEs) or death occurred in the SFI group, whereas 2 CCEs and 1 death occurred in the placebo group. However, no statistical differences in these endpoints were observed between the two groups (P>0.05; Table 7).

### 3.5. Safety Evaluation for SFI

No statistical differences in the occurrence of AEs were observed between the SFI and placebo groups (Table 8). Only 2 ADRs occurred in the SFI group, and the difference from the placebo group was not significant (P>0.05; Table 9). In addition, there were no differences in the laboratory indexes between the SFI and placebo groups (P>0.05; Table 10).

## 4. Discussion

SFI, as a form of TCM, has been long used in clinical practice in China to treat CHF and has achieved favorable outcomes. However, to the best of our knowledge, this study was the first standard clinical trial, i.e., randomized, multicenter, double-blinded, placebo-controlled trial, to explore the safety and efficacy of SFI for treatment of patients in the acute phase of CHF who were simultaneously receiving standard treatments. The major findings included the following: (1) SFI significantly improved the clinical symptoms of these patients; (2) SFI significantly improved cardiac tolerance; and (3) SFI did not induce AEs or ADRs.

With the acceleration of the aging population and advancement in the treatment of human diseases including cardiovascular disorders, the human life expectancy has been extended. However, the number of patients with CHF has also been increasing. Patients with CHF are often hospitalized with acute symptom exacerbation due to various causes. Such patients mainly present with deterioration of cardiac function accompanied by a decline in athletic tolerance. The quest for treatments to alleviate symptoms of CHF during the acute phase of symptom exacerbation has been an active area of research in the clinic. TCM has been used clinically to treat CHF patients with acute symptom exacerbation in China for a long time [16–18], suggesting that TCM may serve as a complementary therapy in combination with standard treatments. In the present study, we evaluated the efficacy of SFI, an old TCM prepared with a new method, for the treatment of CHF patients during the acute exacerbation phase. We found that patients in the SFI treatment group exhibited significantly improved primary endpoints such as NYHA classification and TCM syndrome scores. Mechanistically, the therapeutic effects of SFI may be attributed to its coronary arterial dilation via mediation of NO synthesis/release [19], apoptosis suppression via regulating Blc2 activity [20], and activation of myocardial cell endothelial nitric oxide synthase (eNOS) via targeting PI3K/Akt pathway [21].

NYHA, as a simple, accurate index for evaluating heart function, can reflect the severity of acute heart failure, which has been shown to be closely related to survival [22, 23]. The TCM syndrome score system, which is based on TCM symptoms and signs, is one of the most important and most commonly used indexes for evaluating the effectiveness of a TCM in the treatment of a disease [24]. As a pilot trial, this study used both the NYHA classification and TCM syndrome score as the primary endpoints to examine the efficacy of SFI for treatment of CHF patients. The results showed that SFI treatment in combination with other standard therapies greatly benefited cardiac functional classification and TCM syndrome score compared with the improvements seen in the placebo group, which coincided with improved Lee's heart failure scores and 6MWDs. These findings suggest that SFI treatment may benefit patients in the acute phase of CHF. Notably, no statistical difference in LVEF was observed between the SFI and placebo group, indicating that the improvement of heart functional classification and clinical symptoms by SFI might not be necessarily related to an improvement in cardiac pumping capability. In addition, SFI treatment tended to improve CCEs, but no significant difference was detected between the two groups, which can probably be attributed to the small sample size of our study.

In the present study, we also evaluated the safety of SFI and found no significant differences in the occurrence of ADRs and AEs between the SFI and placebo group, suggesting the appreciable safety of SFI in clinical use.

## 5. Conclusion

In the present study, we demonstrated that SFI treatment in combination with conventional therapy for CHF in the acute phase ameliorated cardiac dysfunction and clinical symptoms, increased the 6MWD, and improved patients' quality of life compared with the conventional therapy alone. We also demonstrated the safety of SFI in clinical use. Future studies with a large cohort are needed to further corroborate our findings and conclusions.

## 6. Limitations

This study was conducted only in some areas, and the observation period was short. Also, we did not see the statistical differences in some endpoint indicators such as CCEs or deaths between these groups. In the future, the study of endpoint events can be carried out sufficiently with greater research funds and manpower.

## Figures and Tables

**Table 1:** Comparison of demographic and baseline clinical characteristics of participants between the SFI and placebo groups.

Characteristic	SFI	Placebo	Statistics	P value
*Basic information*				
Age ($\bar{x}\pm s$, yrs)	68.58±8.42	68.14±8.73	-0.3042	0.7610
Gender				
Male n(%)	42(56.76%)	48(68.57%)	2.1424	0.1433
Female n(%)	32(43.24%)	22(31.43%)		
Weight ($\bar{x}\pm s$, kg)	65.43±13.10	66.26±11.82	0.5744	0.5657
Height ($\bar{x}\pm s$, cm)	164.10±7.30	166.00±6.60	1.63	0.1052
SBP ($\bar{x}\pm s$, mmHg)	132.55±21.89	133.27±19.47	0.2648	0.7912
DBP ($\bar{x}\pm s$, mmHg)	77.26±10.77	77.64±11.04	0.3134	0.7540
Heart rate ($\bar{x}\pm s$, bpm)	86.5±21.6^★^	76.1±20.0	-3.1526	0.0016
*Medical history*				
Disease course of CHF ($\bar{x}\pm s$, yrs)	4.62±6.07	3.76±3.69	-0.4280	0.6687
Disease course of acute exacerbation ($\bar{x}\pm s$, days)	6.08±5.07	6.94±8.27	0.1853	0.8530
History of myocardial infarction n(%)	38(52.05%)	43(61.43%)	1.2785	0.2582
History of arrhythmia n(%)	20(27.03%)	19(27.14%)	0.0002	0.9875
History of hypertension n(%)	51(70.83%)	44(62.86%)	1.0197	0.3126
History of diabetes n(%)	26(35.14%)	16(22.86%)	2.6248	0.1052
*Medication use*				
Antiplatelet n(%)	63 (85.14%)	61 (87.14%)	0.1212	0.7277
Beta-blockers n(%)	36 (48.65%)	44 (62.86%)	2.9412	0.0863
ACE inhibitors n(%)	32 (43.24%)	35 (50.00%)	0.6601	0.4165
ARB inhibitors n(%)	23 (31.08%)	19 (27.14%)	0.2700	0.6033
Statins n(%)	57 (77.03%)	50 (71.43%)	0.5905	0.4422
Nitric acid lipid n(%)	44 (59.46%)	44 (62.86%)	0.1747	0.6759
Ca-antagonist n(%)	18 (24.32%)	17 (24.29%)	0.0000	0.9957
Aldosterone receptor antagonist n(%)	50 (67.57%)	43 (61.43%)	0.5927	0.4414
Diuretics n(%)	45 (60.81%)	35 (50.00%)	1.7027	0.1919
Digoxin n(%)	29 (39.19%)	24 (34.29%)	0.3719	0.5420
*Clinical index*				
TCM syndrome score ($\bar{x}\pm s$)	24.54±7.90	23.37±7.13	-1.1902	0.2340
Lee's heart failure ($\bar{x}\pm s$)	6.36±3.25	5.71±2.92	-1.1787	0.2385
6MWD ($\bar{x}\pm s$, m)	163.28±153.48	185.70±143.30	1.0504	0.2935
LVEF ($\bar{x}\pm s$, %)	38.69±8.52	39.82±8.04	0.7183	0.4726
NYHA classification				
III n(%)	46(62.16%)	51(72.86%)	1.8715	0.1713
IV n(%)	28(37.84%)	19(27.14%)		

^★^P<0.01 compared to control group

**Table 2:** Comparison of NYHA classification between SFI and placebo group.

	Excellent	Valid	Invalid	Worsened	Effective rate	Statistics	P value
SFI (n=74)	18	40	16	0	78.38%	4.9344	0.026
Placebo (n=70)	8	35	27	0	61.43%		

Effective rate was defined as proportion of all patients who experienced an excellent or valid outcome. Similarly, the ineffective rate was defined as the proportion of all patients who experienced an invalid and worsened outcome.

**Table 3:** Comparison of improvement in TCM syndrome score between SFI and placebo groups.

	Excellent	Valid	Invalid	Worsened	Effective rate	Statistics	P value
SFI (n=74)	22	44	7	1	89.19%	16.3459	<0.001
Control (n=70)	14	28	28	0	60.00%		

Effective rate was defined as proportion of all patients who experienced an excellent or valid outcome. Similarly, the ineffective rate was defined as the proportion of all patients who experienced an invalid and worsened outcome.

**Table 4:** Comparison of Lee's heart failure score between SFI and placebo groups.

	Excellent	Valid	Invalid	Worsened	Effective rate	Statistics	P value
SFI (n=74)	23	29	21	1	70.27%	4.9403	0.0262
Placebo (n=69)	15	21	33	0	52.17%		

Effective rate was defined as proportion of all patients who experienced an excellent or valid outcome. Similarly, the ineffective rate was defined as the proportion of all patients who experienced an invalid and worsened outcome.

**Table 5:** Comparison of change in 6MWD (pre-to-post treatment) between SFI and placebo groups.

Endpoint		SFI (n=70)	Placebo (n=69)	P value
6MWD	Posttreatment–pretreatment	113.00±117.55	82.99±127.99	0.0281

**Table 6:** Comparison of change in LVEF (pre-to-post treatment) between SFI and placebo groups.

Endpoint		SFI (n=72)	Placebo (n=67)	P value
LVEF	Post-treatment – pre-treatment	6.58±9.11	4.53±8.21	0.2347

**Table 7:** Comparison of CCEs between SFI and placebo groups.

Endpoint	SFI (n=74)	Placebo (n=70)
CCE	0 (0.00%)	2 (2.86%)
revascularization	0 (0.00%)	2 (2.86%)
Death incident	0 (0.00%)	1 (1.43%)

**Table 8:** Comparison of AEs (top 10) between SFI and placebo groups.

ADE	SFI (n=78)	Placebo (n=79)	All (n=157)
Renal dysfunction	6 (7.69%)	4 (5.06%)	10 (6.37%)
Liver dysfunction	1 (1.28%)	2 (2.53%)	3 (1.91%)
Urinary system infection	5 (6.41%)	3 (3.80%)	8 (5.10%)
Urine protein	1 (1.28%)	1 (1.27%)	2 (1.27%)
Pulmonary infection	1 (1.28%)	0 (0.00%)	1 (0.64%)
Anemia	1 (1.28%)	0 (0.00%)	1 (0.64%)
Hypoglycemia	1 (1.28%)	0 (0.00%)	1 (0.64%)
Chills	1 (1.28%)	0 (0.00%)	1 (0.64%)
Erythra	1 (1.28%)	0 (0.00%)	1 (0.64%)
Diarrhea	0 (0.00%)	1 (1.27%)	1 (0.64%)
Ureteral calculi cut into stone	1 (1.28%)	0 (0.00%)	1 (0.64%)

**Table:** (a) Comparison of ADRs (top 10) between SFI and placebo groups

ADR	SFI (n=78)	Placebo (n=79)	All (n=157)
Chills	1 (1.28%)	0 (0.00%)	1 (0.64%)
Erythra	1 (1.28%)	0 (0.00%)	1 (0.64%)
Total	2 (2.56%)	0 (0.00%)	2 (1.27%)

**Table:** (b) Accessory: list of ADRs

Subject number	Group	ADR symptom	ADR description
006002	SFI	Chill	Chill occurred after injection of 10 ml SFI.
010070	SFI	Erythra	The subject felt itchy skin after 1 day of medication. Skin flushing was seen on the upper parts of the chest and abdomen, the medial buttocks, and both lower limbs. There was scratch, but no break.

**Table 10:** Comparison of incidence of abnormal laboratory indexes between SFI and placebo groups.

Laboratory index	SFI (n=78)	Placebo (n=79)
*Blood routine*		
White blood cell count	12 (16.00%)	9 (11.84%)
Red blood cell count	10 (13.33%)	9 (11.84%)
Hemoglobin	15 (20.00%)	12 (15.79%)
Blood platelet count	11 (14.67%)	8 (10.53%)
*Urine routine*		
Urine protein	6 (11.11%)	10 (16.13%)
Urine sugar	4 (7.41%)	3 (4.84%)
Urine erythrocyte	8 (14.81%)	10 (16.13%)
Urine white blood cell count	11 (20.37%)	8 (12.90%)
*Stool routine*		
Red blood cell	0 (0.00%)	0 (0.00%)
White blood cell	2 (7.14%)	0 (0.00%)
Occult blood	6 (21.43%)	2 (5.13%)
*Hepatic and renal function*		
Alanine transaminase (ALT)	6 (8.45%)	6 (8.57%)
Glutamic oxalacetic transaminase (AST)	7 (9.59%)	5 (6.76%)
Total bilirubin	2 (2.90%)	5 (7.35%)
Urea nitrogen	17 (22.97%)	13 (17.33%)
Creatinine	16 (21.33%)	20 (26.67%)
*Electrolytes*		
K	5 (6.58%)	2 (2.60%)
Na	5 (6.58%)	6 (7.79%)
Cl	3 (3.95%)	5 (6.49%)

## Data Availability

The datasets used and analyzed during the current study are available from the corresponding author on reasonable request.

## Abbreviations

SFI: Shenfu injection
CHF: Chronic heart failure
TCM: Traditional Chinese Medicine
AE: Adverse event
ADR: Adverse drug reaction
NYHA: New York Heart Association
CABG: Coronary artery bypass grafting
PCI: Percutaneous coronary intervention
6MWD: 6-min walking distance
AST: Aspartate aminotransferase
BUN: Blood urea nitrogen
FAS: Full analysis set
PPS: Per-protocol set
GS: Glucose.

## Supplementary Materials

Supplementary Materials Supplemental Table 1: List of participating institutions. Supplemental Table 2: CONSORT 2010 checklist of information to include when reporting a randomised trial. Supplemental Figure 1: Trial flow chart.
